# Poly(alkylidenimine) Dendrimers Functionalized with the Organometallic Moiety [Ru(η^5^-C_5_H_5_)(PPh_3_)_2_]^+^ as Promising Drugs Against *Cisplatin*-Resistant Cancer Cells and Human Mesenchymal Stem Cells

**DOI:** 10.3390/molecules23061471

**Published:** 2018-06-17

**Authors:** Marisol Gouveia, João Figueira, Manuel G. Jardim, Rita Castro, Helena Tomás, Kari Rissanen, João Rodrigues

**Affiliations:** 1 CQM—Centro de Química da Madeira, MMRG, Universidade da Madeira, Campus da Penteada, 9000-390 Funchal, Portugal; marisolgouveia5@gmail.com (M.G.); mgjardim@uma.pt (M.G.J.); ritacastro@uma.pt (R.C.); lenat@uma.pt (H.T.); 2 Department of Chemistry, ScilifeLab, Umeå University, KBC-Building, Linnaeus väg 6, 90736 Umeå, Sweden; joao.figueira@umu.se; 3 Department of Chemistry, University of Jyvaskyla, P.O. Box. 35, FI-40014 Jyväskylä, Finland; kari.t.rissanen@jyu.fi; 4 School of Materials Science and Engineering/Center for Nano Energy Materials, Northwestern Polytechnical University, Xi’an 710072, China

**Keywords:** dendrimers, nanocarriers, metallodrugs, ruthenium, platinum, *cisplatin*, cancer treatment, hMSCs, toxicity, nanomedicine

## Abstract

Here and for the first time, we show that the organometallic compound [Ru(η^5^-C_5_H_5_)(PPh_3_)_2_Cl] (RuCp) has potential to be used as a metallodrug in anticancer therapy, and further present a new approach for the cellular delivery of the [Ru(η^5^-C_5_H_5_)(PPh_3_)_2_]^+^ fragment via coordination on the periphery of low-generation poly(alkylidenimine) dendrimers through nitrile terminal groups. Importantly, both the RuCp and the dendrimers functionalized with [Ru(η^5^-C_5_H_5_)(PPh_3_)_2_]^+^ fragments present remarkable toxicity towards a wide set of cancer cells (Caco-2, MCF-7, CAL-72, and A2780 cells), including cisplatin-resistant human ovarian carcinoma cell lines (A2780*cis*R cells). Also, RuCp and the prepared metallodendrimers are active against human mesenchymal stem cells (hMSCs), which are often found in the tumor microenvironment where they seem to play a role in tumor progression and drug resistance.

## 1. Introduction

Despite their complexity and diversity, oncologic diseases are mainly characterized by the abnormal growth of cells which can gain the potential to invade tissues and disseminate (metastasize) to distant locations in the body [1,2]. According to the U.S. National Cancer Institute, and despite encouraging indicators [3], the number of deaths caused by cancer is expected to increase to 22 million in the next two decades [4], which justifies the current pursuit of new treatments.

The discovery of *cis*-diamminedichloroplatinum (II) (commonly abbreviated as DDP, cisplatin or *cis*Pt) anticancer properties by Rosenberg et al. [5] in 1965, as well as its approval by Food and Drug Administration (FDA) to clinical use in 1978, has triggered the investigation of metal complexes as anticancer chemotherapeutic agents [6,7]. Cisplatin and its second and third-generation platinum drug analogues, *cis*-diammine(1,1-cyclobutanedicarboxylato)platinum(II) (carboplatin) and [(1*R*,2*R*)-cyclohexane-1,2-diamine](ethanedioato-*O*,*O*′)platinum(II) (oxaliplatin), respectively, are the only metal complexes currently used in chemotherapeutic regimes of patients with cancer, being employed alone or in combination with other drugs [8,9,10,11,12,13,14,15]. However, the administration of these platinum-based drugs has been limited due to their substantial adverse side effects (e.g., neurotoxicity) [16,17,18,19,20], incapacity to prevent cancer relapse [20,21], and development of intrinsic or acquired resistance by several types of cancer [16,22,23,24,25,26]. For these reasons, efforts have been made to develop non-platinum metallodrugs with the same objective [6,7,8,9,10,11,13,14,16,23,27,28,29].

Among several metallodrugs that have been explored as anticancer agents, ruthenium compounds have emerged in recent years as promising candidates [27,28,29,30,31]. Some relevant characteristics of ruthenium compounds that have sparked the attention for their application include: (i) the diversity of oxidation states accessible in physiological medium, namely Ru(II), Ru(III) and Ru(IV) [30]; (ii) the slow ligand-exchange kinetics, which can be adjusted by the variation of the nature of the ligands coordinated to the metal [32,33], and (iii) the reduced systemic toxicity. This last property has been associated with the ability of ruthenium to mimic iron in binding several biological molecules, like transferrin and albumin. Thereafter, because cancer cells possess a high number of transferrin receptors on their surface, theoretically, a high level of ruthenium complexes will be delivered preferentially to these cells by transferrin [30,34,35]. Furthermore, it is believed that the inert Ru(III) complexes can be activated to the corresponding cytotoxic forms of Ru(II) in the tumors that possess a reducing environment and, consequently, present a higher selectivity to cancer cells [36].

Many families of ruthenium complexes have been studied against several different types of cancer [28,37,38]. Specifically, the Ru(III) complexes have shown promising results in clinical trials against solid tumors. For example, the imidazolium *trans*-[tetrachlorido(1*H*-imidazole)(*S*-dimethyl sulfoxide)ruthenate(III)] (NAMI-A, an acronym for New Anti-tumour Metastasis Inhibitor A) has concluded the clinical phase I [39] and entered, in combination with gemcitabine (2′,2′-difluoro deoxycytidine), in phase I/II [40]. However, this study is currently suspended due to the toxicity profile and the unclear efficiency of the combination of these drugs [31,40,41]. Other promising Ru(III) compounds are the indazolium *trans*-[tetrachloridobis(1*H*-indazole)ruthenate(III)] (KP1019 or FFC14A) and its analogue sodium *trans*-[tetrachloridobis(1*H*-indazole)ruthenate(III)] (NKP-1339 or IT-139). Both compounds have completed the clinical phase I [42,43,44] but, since NKP-1339 presents higher solubility in water than KP1019, the clinical trials have proceeded only with the former, which is water-soluble [44,45,46]. Also, the incorporation of ruthenium complexes to form multinuclear and supramolecular structures has also been successfully tested on several platforms such as polymers (e.g., polymeric micelles [47]), lipid-based systems [48,49,50,51], and polymer-peptide conjugates [52] with the aim of improving the chemotherapeutic action of these potential drugs.

Among the organoruthenium(II) compounds, the half-sandwich organometallic ruthenium compounds with η^6^-arene [53] or η^5^-cyclopentadienyl [54,55,56] exhibited attracting pharmacological properties to be applied in cancer therapy. In these cases, the aromatic ligand present in the structure of the half sandwich compounds allows the stability and protection of the metal Ru(II) [57,58].

Dendrimers constitute a class of synthetic polymeric macromolecules that possess a hyperbranched structure at the nanosize scale, low polydispersity, and a multifunctional surface [59]. These nanoparticles may be good drug carriers due to the possibility of encapsulating drugs in their interior and/or covalently link them at their surface terminal groups [59,60,61,62]. Besides the potential for carrying multiple drugs and high drug loads, the dendritic multivalency provides increasing interaction with receptors of the therapeutic target [60]. Also, the nanoscale size of the dendrimers allows their selective accumulation in the tumors by the “enhanced permeability and retention” (EPR) effect [61,63].

The incorporation of metal complexes in dendritic scaffolds, thus originating metallodendrimers, can increase the activity and selectivity of drugs based on transition metals [64]. Indeed, metallodendrimers can combine the anticancer potential of metal complexes with the features of the dendrimers as nanocarriers, and were described as having promising cytotoxicity against different cancer cell lines [65,66,67,68,69,70,71,72,73,74,75,76,77,78,79].

In the present work, we started by preparing and characterizing low-generation ruthenium(II) metallodendrimers based on poly(alkylidenimine) dendritic scaffolds peripherally functionalized with the nitrile group and the fragment [Ru(η^5^-C_5_H_5_)(PPh_3_)_2_]^+^. Then, the cytotoxicity of the organometallic compound [Ru(η^5^-C_5_H_5_)(PPh_3_)_2_Cl] (abbreviated by RuCp), the core dendrimers, and the prepared tetrakis-ruthenium dendrimers were tested against five human cancer cell lines: a colorectal adenocarcinoma cell line (Caco-2), an osteosarcoma cell line (CAL-72), a breast adenocarcinoma cell line (MCF-7), and two ovarian carcinoma cell lines (A2780 and A2780*cis*R, the last one resistant to cisplatin), and in healthy human mesenchymal stem cells (hMSCs). In fact, hMSCs are more and more being proposed as a promising target for anticancer drug delivery since many pieces of evidence are arising pointing out their role in tumor development [80,81,82]. hMSCs are known to be recruited into tumors where their action is often described in the literature as pro-tumor, or tumor-supporting, including suppression of the immune response, promotion of angiogenesis, inhibition of apoptosis, stimulation of epithelial to mesenchymal transition and tumor metastasis. Results not only showed that the organometallic moiety [Ru(η^5^-C_5_H_5_)(PPh_3_)_2_]^+^ has an important anticancer activity by itself, but also that its coordination on the periphery of the dendrimers can be used as a successful drug delivery strategy. Furthermore, the present experiments also revealed that both RuCp and the developed dendrimers functionalized with [Ru(η^5^-C_5_H_5_)(PPh_3_)_2_]^+^ fragments presented remarkable toxicity towards cancer cells resistant to cisplatin which is considered a standard in anticancer therapy.

## 2. Results and Discussion

### 2.1. Synthesis and Characterization of [Ru(η^5^-C_5_H_5_)(PPh_3_)_2_]^+^ Functionalized Poly(alkylidenimine) Dendrimers

**Scheme 1:**
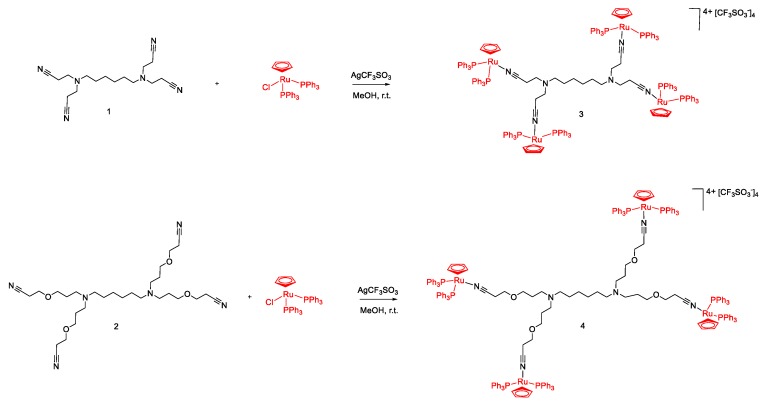
Synthesis of the tetrakis-ruthenium dendrimers **3** and **4**.

Two low generation poly(alkylidenimine) dendrimer cores having nitrile groups at their periphery and distinct in size and flexibility (Scheme 1, dendrimers **1** and **2**) were used to prepare two different [Ru(η^5^-C_5_H_5_)(PPh_3_)_2_]^+^ functionalized poly(alkylidenimine) dendrimers (Scheme 1, metallodendrimers **3** and **4**). The synthesis followed a methodology previously developed by our group [83]. However, because the use of thallium compounds may result in unwanted cytotoxicity, thus hampering the results, in the current work, the prepared compounds were synthesized using a slight modification of the original procedure. AgCF_3_SO_3_ was used as chloride abstractor instead of TlPF_6_. As such, the reaction of a methanolic solution of [Ru(η^5^-C_5_H_5_)(PPh_3_)_2_Cl] and AgCF_3_SO_3_ with the nitrile functionalized poly(alkylidenimine) dendrimers **1** or **2**, at room temperature, afforded the metallodendrimers **3** or **4**, respectively (Scheme 1). These metallodendrimers were isolated as green powders and were characterized by NMR (^1^H, ^31^P, and ^19^F) and infrared (FTIR) spectroscopy, mass spectrometry (MS) and elemental analysis (EA).

**Figure 1:**
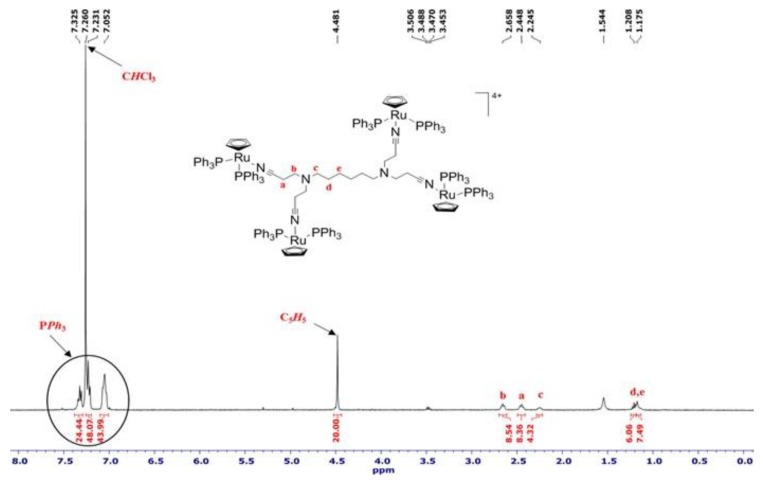
^1^H-NMR spectrum of [{(η^5^-C_5_H_5_)(PPh_3_)_2_Ru}_4_(**1**)][CF_3_SO_3_]_4_ (**3**), in CDCl_3_.

**Figure 2:**
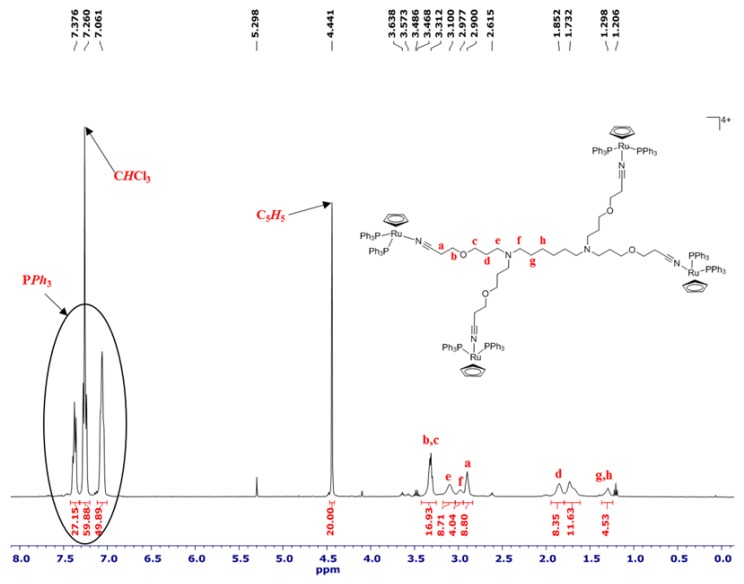
^1^H-NMR spectrum of [{(η^5^-C_5_H_5_)(PPh_3_)_2_Ru}_4_(**2**)][CF_3_SO_3_]_4_ (**4**), in CDCl_3_.

As is evident in the ^1^H-NMR spectra of both tetranuclear metallodendrimers **3** and **4** (Figure 1 and Figure 2, respectively), the presence of only one singlet at 4.48 and 4.44 ppm, respectively, that can be assigned to the protons of the cyclopentadienyl ligand, indicates that the four ruthenium fragments were equivalently coordinated with each dendritic core.

The formation of these compounds was also sustained by the ^31^P-NMR studies (Supplementary Material, Figures S1 and S5) that display a singlet at 41.89 and 41.60 ppm, coming from the resonance of the phosphorus atoms of phosphine ligands, in the spectrum of metallodendrimer **3** and **4**, respectively. The metallodendrimers **3** and **4** presented moderate stability in organic solvents, which was even lower in halogenated solvents, being impossible to obtain ^13^C-NMR spectra for these compounds. The ^19^F-NMR spectra of metallodendrimers **3** and **4** (Supplementary Material, Figures S2 and S6) revealed, respectively, a singlet at −81.78 and at −81.95 ppm that was attributed to the fluorine atoms of the [C*F*_3_SO_3_]^−^ counterions.

The FTIR analysis for both metallodendrimers (**3** and **4**) show, outside the fingerprint zone, similar spectra (Supplementary Material, Figures S3 and S7). The presence of a single nitrile peak shifted to higher wavelengths relative to the position in the free ligand is a clear sign of the formation of the desired compound. Furthermore, the absence of the free v(CN) in the FTIR spectra supports the complete coordination of all nitrile groups present in the dendritic termini. In terms of values, the nitrile stretching band in compound **3** arises at 2271 cm^−1^ while in compound **4** it arises at 2269 cm^−1^. The vibration bands of the [C*F*_3_SO_3_]^−^ counter ion appear in the FTIR spectra around 1274 cm^−1^ and 700 cm^−1^ for metallodendrimer **3**, and ca. 1286 and 697 cm^−1^ for metallodendrimer **4**.

The analysis of the mass spectrum of metallodendrimer **3** (Supplementary Material, Figure S4) shows that the standard fragmentation is consistent with the loss of two counter ions, *m*/*z* = 1694.5096 [M-2CF_3_SO_3_]^2+^, and three counter ions, *m*/*z* = 1081.0131 [M-3CF_3_SO_3_]^3+^, revealing the presence of the desired metallodendrimer. Similar conclusions can be taken from the mass spectrum of metallodendrimer **4** (Supplementary Material, Figure S8) that exhibited the expected isotopic distribution for [M-2CF_3_SO_3_]^2+^ (*m*/*z* = 1810.9692), and [M-3CF_3_SO_3_]^3+^ (*m*/*z* = 1157.9568).

Finally, the results of the elemental analysis confirmed the integrity of the structure of the prepared metallodendrimers **3** and **4** since the calculated theoretical values showed good agreement with those obtained experimentally (data shown in the Section 3).

### 2.2. Biological Activity Assays

**Figure 3:**
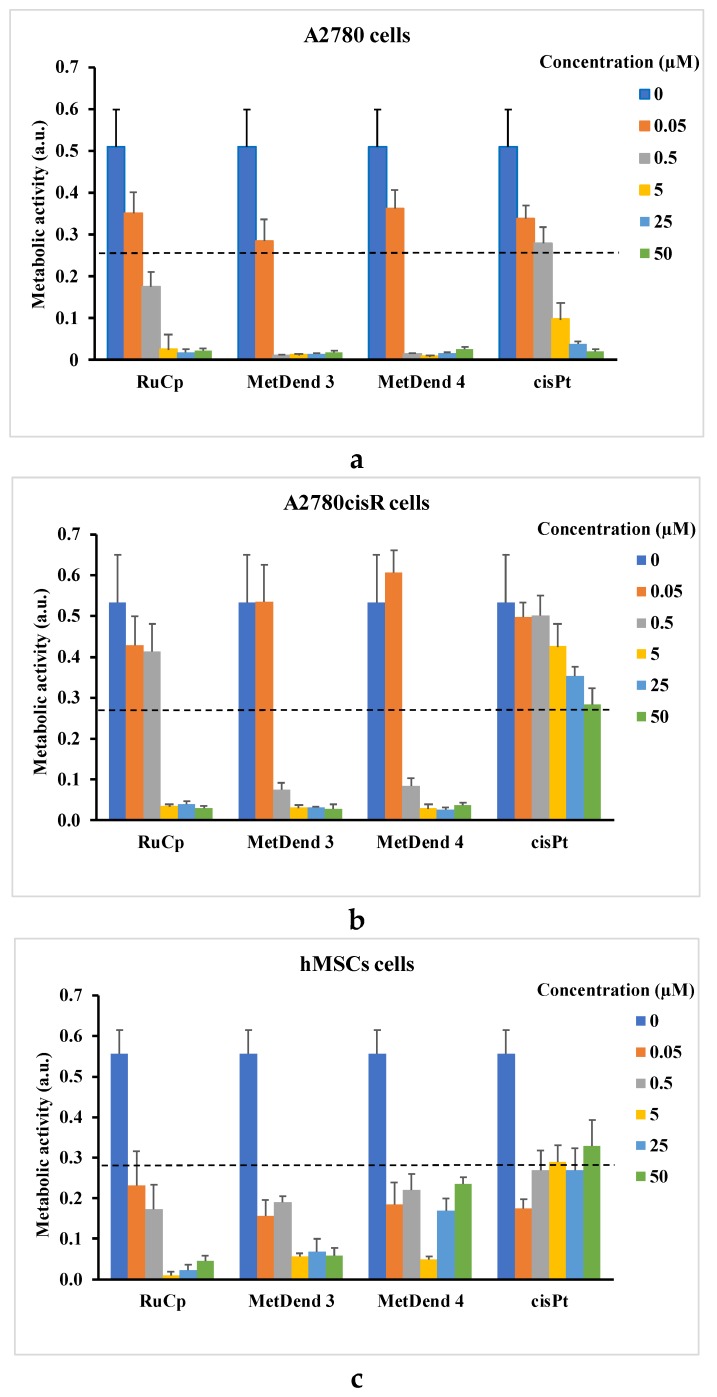
Effect of increasing concentrations of RuCp, metallodendrimers **3** and **4**, and *cis*Pt on the cellular metabolic activity (an indirect measure of cell viability) of (**a**) A2780 and (**b**) A2780*cis*R tumor cell lines, and on (**c**) hMSCs. The dashed line corresponds to 50% of cellular metabolic activity compared to the control. Values are presented as a mean ± standard deviation.

The 3-(4,5-dimethylthiazol-2yl)2,5-diphenyltetrazolium bromide (MTT) assay was used to explore the in vitro cytotoxic potential of the metallodendrimers **3** and **4**. This assay is based on the principle that only cells that are alive are metabolically active, that is, can reduce MTT. For this purpose and in order to cover a broad spectra of cancer types, the response of five human tumor cell lines were investigated, namely a colorectal adenocarcinoma cell line (Caco-2), an osteosarcoma cell line (CAL-72), a breast adenocarcinoma cell line (MCF-7) and two ovarian carcinoma cell lines (A2780 and A2780*cis*R). The cytotoxic effect of the prepared compounds was also evaluated in primary human mesenchymal stem cells (hMSCs). For comparison, the cytotoxicity profile of dendrimers **1** and **2**, [Ru(η^5^-C_5_H_5_)(PPh_3_)_2_Cl] (abbreviated as RuCp), and PPh_3_ were also investigated using the same cell types. Since we also wanted to compare the anticancer potential of metallodendrimers **3** and **4** with that of cisplatin (abbreviated as *cis*Pt), the cytotoxic effect of *cis*Pt was evaluated in A2780 (a cancer cell line sensitive to *cis*Pt) and A2780*cis*R (a cancer cell line resistant to *cis*Pt) cells. In all these assays, the used concentration range for the tested compounds was 0.05 to 50 µM. For RuCp and metallodendrimers **3** and **4**, the concentrations ≥25 µM are only indicative due to solubility issues. The metabolic activity as a function of compound concentration are shown for all compounds in the Supplementary Material (Figures S10 and S11). Figure 3 highlights the data for A2780 and A2780*cis*R cells, as well as for hMSCs.

From Figure S11, it is clear that dendrimer **1** and dendrimer **2** present low cytotoxicity in the range of concentrations studied for all the cancer cell lines. When their concentration is increased, the cellular metabolic activity values remain quite constant. On the contrary, RuCp and metallodendrimers **3** and **4** showed high cytotoxicity which, as expected, generally increased with increasing compound concentration and was cell type-dependent (Figure 3 and Figure S10).

The concentration required to obtain 50% of cell growth inhibition in vitro (IC_50_ value) was determined for these compounds, and as well as for *cis*Pt (when applicable). These results are summarized in Table 1.

Among all cancer cell types studied, the Caco-2 cells were the less sensitive, showing IC_50_ values of 14.7, 3.4 and 3.2 µM for RuCp, metallodendrimer **3** and **4**, respectively. The most sensitive cancer cells were the A2780 cells with IC_50_ values of 0.3, 0.1 and 0.2 µM for RuCp, metallodendrimer **3** and **4**, respectively. The hMSCs that are non-cancer cells were highly sensitive to all ruthenium compounds, and to *cis*Pt also, presenting IC_50_ values lower than the lowest concentration tested (0.05 µM). Interestingly, although these cells seem to be very sensitive to low concentrations of the metallodrugs, an increase in drug concentration above 0.05 µM does not always imply a concomitant decrease in cell viability. This was especially evident for metallodendrimer **4** and *cis*Pt. Likely, either the cellular internalization of these compounds is limited to low concentration values (of the order of magnitude of 0.05 µM) or these cells have internal mechanisms capable of excreting them. Although the role of hMSCs in tumor development is still not well understood and may even involve opposing effects [86,87,88], most of the literature in this subject indicate that they are attracted to cancer sites where they have an overall positive action in tumor progression and metastasis. In some cases, hMSCs even counter-act against anticancer chemotherapeutics [89]. Thus, it is very important to assess the effect of anticancer drugs in these cells. Our results show that RuCp, metallodendrimer **3** and **4** are not only cytotoxic for cancer cell lines, but also for hMSCs, which should contribute to their overall efficiency in anticancer therapeutics.

A significant problem in cancer therapy is the occurrence of drug resistance that requires a continuous search for new therapeutics. All three ruthenium compounds under study presented an anticancer activity towards A2780 cells about one order of magnitude lower than *cis*Pt (IC_50_ = 1.1 µM) that is a drug already under use in the clinic scenario. Importantly, they were also remarkably active against A2780*cis*R cells—the anticancer activity was more than 22 and 166 times higher than *cis*Pt, respectively, for RuCp and both metallodendrimers. For the Caco-2 and MCF-7 cancer cell lines, the anticancer behaviour of the prepared metallodendrimers **3** and **4** was ca. 3 times better than *cis*Pt. Thus, RuCp, metallodendrimers **3** and **4** could be good candidates for the therapy of *cis*Pt resistant tumors. As far as we know and despite being a compound widely used in organometallic chemistry as a starting material for different applications, including metallodrugs [55,56,57,59], the anticancer activity of RuCp was never reported in the literature and particularly referred as an active compound against tumors resistant to *cis*Pt.

The IC_50_ values of RuCp were always higher than those of metallodendrimers that possess four coordinated [Ru(η^5^-C_5_H_5_)(PPh_3_)_2_]^+^ organometallic fragments. Possibly, for the metallodendrimers, the mechanism of drug cytotoxicity involves the release (de-coordination) of ruthenium containing fragments from the organic cores. Therefore, the organic dendrimer core serves as a vehicle for the cellular delivery of several [Ru(η^5^-C_5_H_5_)(PPh_3_)_2_]^+^ “toxic” fragments. Furthermore, we previously showed by ^31^P NMR spectroscopy that these metallodendrimers could suffer a degradation process at 37 °C [90]. Since the organic cores and PPh_3_ did not show relevant toxicity by themselves (Supplementary Material, Figure S11), [Ru(η^5^-C_5_H_5_)(PPh_3_)_2_]^+^ should certainly be among the metallodendrimer degradation products. Also, the de-coordination of PPh_3_ was not supported by NMR studies.

An additional observation of the present work was that the difference in the structure of the core (dendrimer **2** is more extensive and flexible than dendrimer **1**) had no especial impact over the cytotoxic behavior of the metallodendrimers which were both strongly cytotoxic.

**Figure A1:**
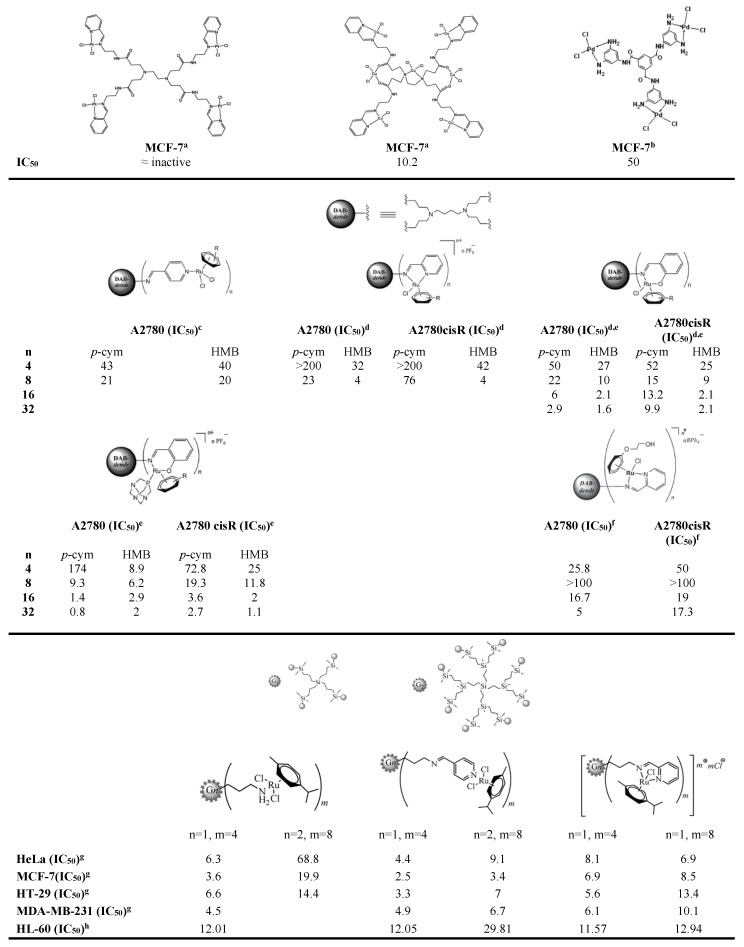
IC_50_ values (in µM) of some metallodendrimers reported in the literature. (a) [68]; (b) [71]; (c) [67]; (d) [69]; (e) [72]; (f) [77]; (g) [78] and (h) [79].

Despite the usual differences between reported experimental conditions, the metallodendrimers **3** and **4** present IC_50_ values lower than other metallodendrimers reported in the literature, including high generation metallodendrimers (see some examples in Appendix A, Figure A1). For instance, by comparison with the fourth-generation of the chelating *N*,*O*-ruthenium(II)-arene-PTA metallodendrimers [72], our compounds were found to be 3.7 to 20 times more active against A2780 and A2780*cis*R cells. Interesting is also to compare, our IC_50_ values for metallodendrimers **3** and **4** with non-dendrimeric systems containing ruthenium complexes. For example, our simple metallodendrimers **3** and **4**, with four coordinated [Ru(η^5^-C_5_H_5_)(PPh_3_)_2_]^+^ organometallic fragments, when compared with cyclic peptide–polymer self-assembling nanotubes conjugated to ruthenium(II)-arene complex [Ru(η^6^-p-cymene)Cl_2_(pta)] (RAPTA-C, a very active drug against metastases in vivo), were about 74 times more active against A2780 and A2780*cis*R cells [52]. They were also 65 times more cytotoxic than NAMI-A block copolymer micelles against the A2780 cancer cell line [47]. Even with the necessary reservations, the in vitro results obtained for metallodendrimers **3** and **4**, with the [Ru(η^5^-C_5_H_5_)(PPh_3_)_2_]^+^ organometallic fragment, compared with the published metallodendrimers or other multinuclear and supramolecular structures involving ruthenium-complexes, seem to be very promising and worthy of further study.

## 3. Materials and Methods

### 3.1. General Remarks

Unless otherwise noted, chemicals were used as received. The solvents diethyl ether (VWR), and dichloromethane (HPLC grade, Fisher Scientific, Hampton, NH, USA,) were distilled from sodium/benzophenone ketyl and calcium hydride (ACROS/Thermo Fisher Scientific, Waltham, MA USA), respectively, under a nitrogen atmosphere before use. Absolute methanol (Sigma-Aldrich, St. Louis, MO, USA) and benzene (PanReac, Barcelona, Spain) were degassed before use by bubbling with nitrogen. Dimethylsulfoxide (DMSO) for biological assays and AgCF_3_SO_3_ were purchased from VWR (Radnor, PA, USA) and ACROS, respectively. Deuterated solvents (CDCl_3_, DMSO-D_6_, D_2_O) were purchased from EURISO-TOP (Saint-Aubin, France).

All reactions and manipulations involved in the preparation of the dendrimers [N≡C(CH_2_)_2_]_2_N(CH_2_)_6_N[(CH_2_)_2_C≡N]_2_ (**1**) and [N≡C(CH_2_)_2_O(CH_2_)_3_]_2_N(CH_2_)_6_N[(CH_2_)_3_O(CH_2_)_2_C≡N]_2_ (**2**), and the metallodendrimers **3** and **4** were executed under a dry nitrogen atmosphere by applying standard Schlenk-tube techniques. The starting materials [Ru(η^5^-C_5_H_5_)(PPh_3_)_2_Cl] [91] and the dendrimers **1** and **2** were prepared by following published methods [83].

### 3.2. Physical Measurements

^1^H (400 MHz), ^13^C{1H} (100 MHz), ^31^P{1H} (161 MHz) and ^19^F{1H} (376 MHz) NMR spectra were recorded on an Avance II^+^ 400 spectrometer (Bruker, Wissembourg, France) at 299 K (probe temperature). The chemical shifts are reported in parts per million (δ, ppm) and referenced to residual solvent peaks for ^1^H (CDCl_3_: δ = 7.26 ppm). The ^31^P- and ^19^F-NMR were referenced to the external aqueous solution of 85% H_3_PO_4_ and KF at 0.5 M, respectively in CDCl_3_ (or in a mixture of DMSO-D_6_/D_2_O for PPh_3_ spectra—see Supplementary Material, Figure S9). The IR spectra were measured on an Avatar 360 FTIR (Nicolet, Thermo Scientific, Waltham, MA, USA) in KBr pellets; only significant bands are mentioned in the text. The mass spectra (ESI-TOF) were recorded with a Micromass LCT mass spectrometer (Waters, Milford, MA, USA). Elemental analyses (C, H, N) were performed in a VariolEL instrument from Elementar Analysensysteme (Langenselbold, Germany). In the processing of the elemental analysis results of compound **3** and **4**, the theoretical values were calculated taking into account the addition of dichloromethane molecules since their presence is observed in the ^1^H-NMR spectra of both compounds. This situation arises from the inclusion of solvent molecules and/or inorganic salts in the dendritic structures during the isolation of the compound by precipitation.

### 3.3. Synthesis

#### 3.3.1. Synthesis of [{(η^5^-C_5_H_5_)(PPh_3_)_2_Ru}_4_(1)][CF_3_SO_3_]_4_ (**3**)

The compound was prepared by reaction of [Ru(η^5^-C_5_H_5_)(PPh_3_)_2_Cl] (0.32 g, 0.44 mmol) with compound **1** (0.04 g, 0.11 mmol) and AgCF_3_SO_3_ (0.15 g, 0.58 mmol) in methanol (59 mL). The yellow-green suspension was stirred for 76 h at room temperature and protected from light. After the reaction, the resulting brown suspension was filtered, and the solid was extracted with dichloromethane. Then, the addition of diethyl ether to the resulting solution afford the precipitation of the desired compound. The solvent was removed, and the product was washed several times with diethyl ether and dried in under vacuum, resulting in a pale green powder. Yield: 0.14 g (35%). ^1^H-NMR (CDCl_3_): δ = 7.40–6.90 (m, 24H + 48H+ 48H, P*Ph*_3_), 4.48 (s, 20 H, C_5_*H*_5_), 2.66 (br., 8H), 2.45 (br., 8H), 2.24 (br., 4H), 1.18 (br, 8H) ppm. ^31^P-NMR (CDCl_3_): δ = 41.89 (s, *P*Ph_3_) ppm. ^19^F-NMR (CDCl_3_): δ = −81.78 ppm. FTIR (KBr): ῦ = 2271 (ν_CN_) and 1274 (ν_CF3SO3_) cm^−1^. TOF-MS(ESI+): *m*/*z* = 1694.5096 [M-2CF_3_SO_3_]^2+^, 1081.0131[M-3CF_3_SO_3_]^3+^. EA(%): C_186_H_168_F_12_N_6_O_12_P_8_Ru_4_S_4_^.^1.3CH_2_Cl_2_ (3715.98): calcd. C 59.23, H 4.53, N 2.21; found C 59.21, H 4.54, N 2.20.

#### 3.3.2. Synthesis of [{(η^5^-C_5_H_5_)(PPh_3_)_2_Ru}_4_(2)][CF_3_SO_3_]_4_ (**4**)

Compound **4** was prepared by reaction of [Ru(η^5^-C_5_H_5_)(PPh_3_)_2_Cl] (0.46 g, 0.63 mmol), compound **2** (0.07 g, 0.13 mmol) and AgCF_3_SO_3_ (0.17 g, 0.66 mmol) in methanol (42 mL). The resulting brown suspension was stirred for 66 h at room temperature under protection from light. The reaction mixture was filtered and dried under vacuum. Then, the yellow-brown solid was extracted with dichloromethane, dried and washed with diethyl ether and benzene. The dark green product was dissolved in dichloromethane, and the resulting solution was filtered and then concentrated under reduced pressure. The addition of diethyl ether to the previous solution originated the formation of dark green oil. This oil was isolated by removing the solvent and then washed with diethyl ether giving a bright green powder. Yield: 0.13 g (25%). ^1^H-NMR (CDCl_3_): δ = 7.50–7.00 (m, 24H + 48H + 48H, P*Ph*_3_), 4.44 (s, 20H, C_5_*H*_5_), 3.31 (br., 8H + 8H), 3.10 (br., 8H), 2.98 (br., 4H), 2.90 (br, 8H), 1.85 (br., 8H), 1.30 (br., 4H) ppm. ^31^P-NMR (CDCl_3_): δ = 41.60 (s, *P*Ph_3_) ppm. ^19^F-NMR (CDCl_3_): δ= −81.95 ppm. FTIR (KBr): ῦ = 2269 (νCN), 1286 and 697 (νCF_3_SO_3_) cm^−1^. TOF-MS(ESI+): *m*/*z* = 1810.9692 [M-_2_CF_3_SO_3_]^2+^ and 1157.9568 [M-3CF_3_SO_3_]^3+^. ES(%): C_198_H_192_F_12_N_6_O_16_P_8_Ru_4_S_4_^.^3CH_2_Cl_2_ (4174.8): calcd. C 57.83, H 4.78, N 2.01; found C 57.79, H 4.79, N 2.04.

### 3.4. Cytotoxicity Studies

#### 3.4.1. Cell Culture

The human cell lines Caco-2, CAL-72, and MCF-7 were purchased from German Collection of Microorganisms and Cell Cultures (DSMZ, Braunschweig, Germany), whereas A2780 and A2780*cis*R human cell lines were obtained from European Collection of Cell Cultures (ECACC, Salisbury, UK). The hMSCs were obtained from patient trabecular bone samples collected during surgical interventions performed after traumatic events (the only bone that would have been discarded was used). For this, the approval of the Ethics Committee of Dr. Nélio Mendonça Hospital (Funchal, Madeira main hospital) was obtained.

Caco-2 cells were grown in MEM medium supplemented with 20% (*v*/*v*) fetal bovine serum (FBS), 1% (*v*/*v*) nonessential amino acids (NEAA, from 100× ready-to-use stock solution) and 1% (*v*/*v*) antibiotic-antimycotic (AA, from 100× solution). CAL-72 cells were grown in DMEM medium enriched with 10% (*v*/*v*) FBS, 1% (*v*/*v*) insulin-transferrin-sodium selenite (ITS, from 100× solution), 2 mM L-glutamine and 1% antibiotic-antimycotic (AA, from 100× solution). MCF-7 cells were grown in RPMI 1640 medium supplemented with 20% (*v*/*v*) FBS, 1% (*v*/*v*) nonessential amino acids (NEAA, from 100× solution), 1 mM sodium pyruvate, 3.3 µg/mL human insulin and 1% (*v*/*v*) antibiotic-antimycotic (AA, from 100× solution). A2780 and A2780*cis*R were grown in RPMI 1640 medium supplemented with 10% (*v*/*v*) FBS, 2 mM L-glutamine and 1% (*v*/*v*) antibiotic-antimycotic (AA, from 100× solution). The hMSCs were grown in α-MEM medium supplemented with 10% (*v*/*v*) FBS and 1% (*v*/*v*) antibiotic-antimycotic (AA, from 100× solution). All cells were maintained at 37 °C in an incubator under a humidified atmosphere containing 5% CO_2_.

#### 3.4.2. Cell Viability Evaluation

The cell viability was indirectly determined by the MTT assay, which measures the mitochondrial dehydrogenase activity as an indication of cell survival.

Cells were counted using a hemocytometer and were seeded in 96-well plates by the addition of 100 µL of cell solution per well at the following cellular densities: 2 × 10^3^ (Caco-2 and CAL-72), 4.2 × 10^3^ (MCF-7), 5 × 10^3^ (A2780 and A2780*cis*R) and 4.8 × 10^3^ (hMSCs). The tested compounds were prepared in a stock solution of DMSO and serially diluted, in the same solvent, to different concentrations. Then, the resulting solutions were diluted in complete culture medium to the desired concentrations with a final DMSO concentration of 0.5% (*v*/*v*).

After 24 h of preincubation of the cells plates at 37 °C and 5% CO_2_, the medium was aspirated, and 100 µL/well of complete medium containing the compound under test was added to the cells. Control experiments were done with cells cultured in complete culture medium with 0.5% (*v*/*v*) of DMSO. All tested conditions were accomplished in replicates of eight. All these culture plates were incubated for 72 h at 37 °C and 5% CO_2_. After this period, the culture medium was aspirated and 100 µL of culture medium solution with 10% (*v*/*v*) of MTT solution (5 mg/mL) was added to each well. Then, after 3 to 4 h of incubation of the plates with MTT, the culture medium was aspirated, and DMSO was added to dissolve the formed purple formazan crystals. The absorbance reading was performed at 550 nm in a microplate reader (Victor3 1420, Perkin Elmer, Waltham, MA, USA) and the cell viability was determined. The concentration that inhibited 50% of the cellular metabolic activity (IC_50_) was calculated by linear interpolation between the two experimental points closer to the point correspondent to 50% of the cellular metabolic activity shown by the control.

## 4. Conclusions

In summary, low-generation ruthenium (II) metallodendrimers based on two nitrile poly(alkylidenimine) dendritic scaffolds (differing in size and flexibility) and containing at the periphery the organometallic fragment [Ru(η^5^-C_5_H_5_)(PPh_3_)_2_]^+^ were synthesized and characterized. The core dendrimers **1** and **2** presented low cytotoxicity on all the cancer cell lines studied. Opposite behavior was observed for the prepared metallodendrimers and compound [Ru(η^5^-C_5_H_5_)(PPh_3_)_2_Cl] that revealed, a high anticancer activity towards different cancer cell lines (Caco-2, CAL-72, and MCF-7) and a high inhibitory effect on the viability of hMSCs in vitro (cells that are believed to be implicated in tumor progression). The latter compounds also presented high activity against cell lines resistant to *cis*Pt (A2780*cis*R), with its anticancer activity being 22 and 166 times more higher than *cis*Pt, respectively, for RuCp and both metallodendrimers, tackling an important and real problem in the context of anticancer therapy. Also, the IC_50_ values of the prepared dendrimers are lower than other metallodendrimers reported in the literature, and 3.7 to 20 times more active against A2780 and A2780*cis*R cells.

With this work and to the best of our knowledge for the first time, we present evidences of the potential of an old organometallic complex, the [Ru(η^5^-C_5_H_5_)(PPh_3_)_2_Cl], as an anticancer drug, but also that the toxic fragment [Ru(η^5^-C_5_H_5_)(PPh_3_)_2_]^+^ could be delivered into cells using nitrile poly(alkylidenimine) dendritic scaffolds. We hypothesize that the delivery of these “new” drugs directly in the tumor site (local delivery) or, in the alternative, their association with nanomaterials for targeted and controlled delivery into tumors [92,93], would be the right strategy for their use in cancer therapy. Indeed, the high toxicity of these compounds towards different cancer cells and hMSCs can potentially be exploited but like happens with other anticancer drugs, undesired off-target effects must be avoided. Currently, we are focused on the design of nanocarriers dendrimers based on the targeted delivery of RuCp, as well as on the study of the possible mechanisms underlying their anticancer activity and pharmacokinetic behavior.

## Figures and Tables

**Table 1:** IC_50_ values (in µM) of RuCp, metallodendrimers **3** and **4**, and *cis*Pt observed for Caco-2, CAL 72, MCF-7, A2780 and A2780*cis*R cancer cells, as well as for hMSCs, after 72 h of exposition to the compounds.

	Cell Type (IC_50_ Values in µM)^1^					
Compound	Caco-2	CAL-72	MCF-7	A2780	A2780*cis*R	hMSCs
RuCp	14.7	2.4	4.4	0.3	2.3	<0.05
Metallodendrimer **3**	3.4	0.6	2.5	0.1	0.3	<0.05
Metallodendrimer **4**	3.2	1.4	3.0	0.2	0.3	<0.05
*cis*Pt	8.9 [84]	- ^2^	7.6 [85]	1.1	>50	<0.05

^1^ The IC_50_ values were determined by linear interpolation between the two nearest neighbour experimental points. The standard deviation was always less than 10% of the IC_50_ value. ^2^ Not reported in the literature.

## Supplementary Materials

The following are available online. Characterization data and Cytotoxicity assays of synthesized and studied compounds.

## References

[1] Ruddon R.W. (2007). Cancer Biology.

[2] Hesketh R. (2013). Introduction to Cancer Biology: A Concise Journey from Epidemiology Through Cell and Molecular Biology to Treatment and Prospects.

[3] Cronin K.A., Lake A.J., Scott S., Sherman R.L., Noone A., Howlader N., Henley S.J., Anderson R.N., Firth A.U., Ma J. (2018). Annual Report to the Nation on the Status of Cancer, part I: National cancer statistics. Cancer.

[4] National Cancer Institute—Cancer Statistics. https://www.cancer.gov/about-cancer/understanding/statistics.

[5] Rosenberg B., Van Camp L., Krigas T. (1965). Inhibition of Cell Division in Escherichia Coli by Electrolysis Products from a Platinum Electrode. Nature.

[6] Trudu F., Amato F., Vaňhara P., Pivetta T., Peña-Méndez E.M., Havel J. (2015). Coordination Compounds in Cancer: Past, Present and Perspectives. J. Appl. Biomed..

[7] Monneret C. (2011). Platinum Anticancer Drugs. From Serendipity to Rational Design. Ann. Pharm. Françaises.

[8] Besse B., Adjei A., Baas P., Meldgaard P., Nicolson M., Paz-Ares L., Reck M., Smit E.F., Syrigos K., Stahel R. (2014). 2nd ESMO Consensus Conference on Lung Cancer: Non-Small-Cell Lung Cancer First-Line/second and Further Lines of Treatment in Advanced Disease. Ann. Oncol..

[9] Gahart B.L., Nazareno A.R. (2014). 2015 Intravenous Medications: A Handbook for Nurses and Health Professionals; Intravenous Medications.

[10] Partridge A.H., Rumble R.B., Carey L.A., Come S.E., Davidson N.E., Di Leo A., Gralow J., Hortobagyi G.N., Moy B., Yee D. (2014). Chemotherapy and Targeted Therapy for Women With Human Epidermal Growth Factor Receptor 2—Negative (or Unknown) Advanced Breast Cancer: American Society of Clinical Oncology Clinical Practice Guideline. J. Clin. Oncol..

[11] Hanna N.H., Einhorn L.H. (2014). Testicular Cancer—Discoveries and Updates. N. Engl. J. Med..

[12] Ghosn M., Kourie H.R., El Rassy E., Chebib R., El Karak F., Hanna C., Nasr D. (2015). Optimum Chemotherapy for the Management of Advanced Biliary Tract Cancer. World J. Gastroenterol..

[13] Pignata S., Scambia G., Katsaros D., Gallo C., Pujade-Lauraine E., De Placido S., Bologna A., Weber B., Raspagliesi F., Panici P.B. (2014). Carboplatin plus Paclitaxel Once a Week versus Every 3 Weeks in Patients with Advanced Ovarian Cancer (MITO-7): A Randomised, Multicentre, Open-Label, Phase 3 Trial. Lancet Oncol..

[14] Liao B.-C., Shao Y.-Y., Chen H.-M., Shau W.-Y., Lin Z.-Z., Kuo R.N., Lai C.-L., Chen K.-H., Cheng A.-L., Yang J.C.-H. (2015). Comparative Effectiveness of First-Line Platinum-Based Chemotherapy Regimens for Advanced Lung Squamous Cell Carcinoma. Clin. Lung Cancer.

[15] Smith J.J., Garcia-Aguilar J. (2015). Advances and Challenges in Treatment of Locally Advanced Rectal Cancer. J. Clin. Oncol..

[16] Rabik C.A., Dolan M.E. (2007). Molecular Mechanisms of Resistance and Toxicity Associated with Platinating Agents. Cancer Treat. Rev..

[17] Langer T., am Zehnhoff-Dinnesen A., Radtke S., Meitert J., Zolk O. (2013). Understanding Platinum-Induced Ototoxicity. Trends Pharmacol. Sci..

[18] Avan A., Postma T.J., Ceresa C., Avan A., Cavaletti G., Giovannetti E., Peters G.J. (2015). Platinum-Induced Neurotoxicity and Preventive Strategies: Past, Present, and Future. Oncologist.

[19] Krüger K., Thomale J., Stojanović N., Osmak M., Henninger C., Bormann S., Fritz G. (2015). Platinum-Induced Kidney Damage: Unraveling the DNA Damage Response (DDR) of Renal Tubular Epithelial and Glomerular Endothelial Cells Following Platinum Injury. Biochim. Biophys. Acta Mol. Cell Res..

[20] McWhinney S.R., Goldberg R.M., McLeod H.L. (2009). Platinum Neurotoxicity Pharmacogenetics. Mol. Cancer Ther..

[21] Martin L.P., Hamilton T.C., Schilder R.J. (2008). Platinum Resistance: The Role of DNA Repair Pathways. Clin. Cancer Res..

[22] Davis A., Tinker A.V., Friedlander M. (2014). “Platinum Resistant” Ovarian Cancer: What Is It, Who to Treat and How to Measure Benefit?. Gynecol. Oncol..

[23] Moutinho C., Martinez-Cardús A., Santos C., Navarro-Pérez V., Martínez-Balibrea E., Musulen E., Carmona F.J., Sartore-Bianchi A., Cassingena A., Siena S. (2014). Epigenetic Inactivation of the BRCA1 Interactor SRBC and Resistance to Oxaliplatin in Colorectal Cancer. J. Natl. Cancer Inst..

[24] Siddik Z.H. (2003). Cisplatin: Mode of Cytotoxic Action and Molecular Basis of Resistance. Oncogene.

[25] Jacobsen C., Honecker F. (2015). Cisplatin Resistance in Germ Cell Tumours: Models and Mechanisms. Andrology.

[26] Horibe S., Matsuda A., Tanahashi T., Inoue J., Kawauchi S., Mizuno S., Ueno M., Takahashi K., Maeda Y., Maegouchi T. (2015). Cisplatin Resistance in Human Lung Cancer Cells Is Linked with Dysregulation of Cell Cycle Associated Proteins. Life Sci..

[27] Bergamo A., Gaiddon C., Schellens J.H., Beijnen J.H., Sava G. (2012). Approaching Tumour Therapy beyond Platinum Drugs: Status of the Art and Perspectives of Ruthenium Drug Candidates. J. Inorg. Biochem..

[28] Zeng L., Gupta P., Chen Y., Wang E., Ji L., Chao H., Chen Z.-S. (2017). The Development of Anticancer Ruthenium(II) Complexes: From Single Molecule Compounds to Nanomaterials. Chem. Soc. Rev..

[29] Lazarević T., Rilak A., Bugarčić Ž.D. (2017). Platinum, palladium, gold and ruthenium complexes as anticancer agents: Current clinical uses, cytotoxicity studies and future perspectives. Eur. J. Med. Chem..

[30] Allardyce C.S., Dyson P.J. (2001). Ruthenium in Medicine: Current Clinical Uses and Future Prospects. Platin. Met. Rev..

[31] Alessio E., Guo Z. (2017). Metal Anticancer Complexes—Activity, Mechanism of Action, Future Perspectives. Eur. J. Inorg. Chem..

[32] Reedijk J. (2008). Metal-Ligand Exchange Kinetics in Platinum and Ruthenium Complexes. Platin. Met. Rev..

[33] Bruijnincx P.C.A., Sadler P.J. (2009). Controlling Platinum, Ruthenium, and Osmium Reactivity for Anticancer Drug Design. Adv. Inorg. Chem..

[34] Pongratz M., Schluga P., Jakupec M.A., Arion V.B., Hartinger C.G., Allmaier G., Keppler B.K. (2004). Transferrin Binding and Transferrin-Mediated Cellular Uptake of the Ruthenium Coordination Compound KP1019, Studied by Means of AAS, ESI-MS and CD Spectroscopy. J. Anal. At. Spectrom..

[35] Guo W., Zheng W., Luo Q., Li X., Zhao Y., Xiong S., Wang F. (2013). Transferrin Serves As a Mediator to Deliver Organometallic Ruthenium(II) Anticancer Complexes into Cells. Inorg. Chem..

[36] Antonarakis E.S., Emadi A. (2010). Ruthenium-Based Chemotherapeutics: Are They Ready for Prime Time?. Cancer Chemother. Pharmacol..

[37] Ang W.H., Dyson P.J. (2006). Classical and Non-Classical Ruthenium-Based Anticancer Drugs: Towards Targeted Chemotherapy. Eur. J. Inorg. Chem..

[38] Levina A., Mitra A., Lay P.A. (2009). Recent Developments in Ruthenium Anticancer Drugs. Metallomics.

[39] Rademaker-Lakhai J.M., van den Bongard D., Pluim D., Beijnen J.H., Schellens J.H.M. (2004). A Phase I and Pharmacological Study with Imidazolium-Trans-DMSO-Imidazole-Tetrachlororuthenate, a Novel Ruthenium Anticancer Agent. Clin. Cancer Res..

[40] Leijen S., Burgers S., Baas P., Pluim D., Tibben M., van Werkhoven E., Alessio E., Sava G., Beijnen J., Schellens J.M. (2015). Phase I/II Study with Ruthenium Compound NAMI-A and Gemcitabine in Patients with Non-Small Cell Lung Cancer after First Line Therapy. Investig. New Drugs.

[41] Bergamo A., Sava G. (2015). Linking the Future of Anticancer Metal-Complexes to the Therapy of Tumour Metastases. Chem. Soc. Rev..

[42] Hartinger C.G., Jakupec M.A., Zorbas-Seifried S., Groessl M., Egger A., Berger W., Zorbas H., Dyson P.J., Keppler B.K. (2008). KP1019, A New Redox-Active Anticancer Agent—Preclinical Development and Results of a Clinical Phase I Study in Tumor Patients. Chem. Biodivers..

[43] Lentz F., Drescher A., Lindauer A., Henke M., Hilger R.A., Hartinger C.G., Scheulen M.E., Dittrich C., Keppler B.K., Jaehde U. (2009). Research-EWIV, in collaboration with C. E. S. for A. D. Pharmacokinetics of a Novel Anticancer Ruthenium Complex (KP1019, FFC14A) in a Phase I Dose-Escalation Study. Anticancer Drugs.

[44] Trondl R., Heffeter P., Kowol C.R., Jakupec M.A., Berger W., Keppler B.K. (2014). NKP-1339, the First Ruthenium-Based Anticancer Drug on the Edge to Clinical Application. Chem. Sci..

[45] Chellan P., Sadler P.J. (2015). The Elements of Life and Medicines. Philos. Trans. Ser. A Math. Phys. Eng. Sci..

[46] Dömötör O., Hartinger C., Bytzek A., Kiss T., Keppler B., Enyedy E. (2013). Characterization of the Binding Sites of the Anticancer ruthenium(III) Complexes KP1019 and KP1339 on Human Serum Albumin via Competition Studies. JBIC J. Biol. Inorg. Chem..

[47] Blunden B.M., Rawal A., Lu H., Stenzel M.H. (2014). Superior Chemotherapeutic Benefits from the Ruthenium-Based Anti-Metastatic Drug NAMI-A through Conjugation to Polymeric Micelles. Macromolecules.

[48] Montesarchio D., Mangiapia G., Vitiello G., Musumeci D., Irace C., Santamaria R., D’Errico G., Paduano L. (2013). A new design for nucleolipid-based Ru(III) complexes as anticancer agents. Dalton Trans..

[49] Vitiello G., Luchini A., D’Errico G., Santamaria R., Capuozzo A., Irace C., Montesarchio D., Paduano L. (2015). Cationic Liposomes as Efficient Nanocarriers for the Drug Delivery of an Anticancer Cholesterol-Based Ruthenium Complex. J. Mater. Chem. B.

[50] Irace C., Misso G., Capuozzo A., Piccolo M., Riccardi C., Luchini A., Caraglia M., Paduano L., Montesarchio D., Santamaria R. (2017). Antiproliferative effects of ruthenium-based nucleolipidic nanoaggregates in human models of breast cancer in vitro: Insights into their mode of action. Sci. Rep..

[51] Riccardi C., Musumeci D., Irace C., Paduano L., Montesarchio D. (2017). Ru^III^ Complexes for Anticancer Therapy: The Importance of Being Nucleolipidic. Eur. J. Org. Chem..

[52] Blunden B.M., Chapman R., Danial M., Lu H., Jolliffe K.A., Perrier S., Stenzel M.H. (2014). Drug Conjugation to Cyclic Peptide-Polymer Self-Assembling Nanotubes. Chem. Eur. J..

[53] Suss-Fink G. (2010). Arene Ruthenium Complexes as Anticancer Agents. Dalton Trans..

[54] Garcia M.H., Morais T.S., Florindo P., Piedade M.F., Moreno V., Ciudad C., Noe V. (2009). Inhibition of Cancer Cell Growth by ruthenium(II) Cyclopentadienyl Derivative Complexes with Heteroaromatic Ligands. J. Inorg. Biochem..

[55] Tomaz A.I., Jakusch T., Morais T.S., Marques F., de Almeida R.F.M., Mendes F., Enyedy É.A., Santos I., Pessoa J.C., Kiss T. (2012). [Ru^II^(η^5^-C_5_H_5_)(bipy)(PPh_3_)]^+^, a Promising Large Spectrum Antitumor Agent: Cytotoxic Activity and Interaction with Human Serum Albumin. J. Inorg. Biochem..

[56] Morais T.S., Santos F.C., Jorge T.F., Côrte-Real L., Madeira P.J.A., Marques F., Robalo M.P., Matos A., Santos I., Garcia M.H. (2014). New Water-Soluble ruthenium(II) Cytotoxic Complex: Biological Activity and Cellular Distribution. J. Inorg. Biochem..

[57] Peacock A.F.A., Sadler P.J. (2008). Medicinal Organometallic Chemistry: Designing Metal Arene Complexes as Anticancer Agents. Chem. An Asian J..

[58] Morais T.S., Silva T.J.L., Marques F., Robalo M.P., Avecilla F., Madeira P.J.A., Mendes P.J.G., Santos I., Garcia M.H. (2012). Synthesis of Organometallic ruthenium(II) Complexes with Strong Activity against Several Human Cancer Cell Lines. J. Inorg. Biochem..

[59] Kesharwani P., Jain K., Jain N.K. (2014). Dendrimer as Nanocarrier for Drug Delivery. Prog. Polym. Sci..

[60] Nanjwade B.K., Bechra H.M., Derkar G.K., Manvi F.V., Nanjwade V.K. (2009). Dendrimers: Emerging Polymers for Drug-Delivery Systems. Eur. J. Pharm. Sci..

[61] Pérez-Herrero E., Fernández-Medarde A. (2015). Advanced Targeted Therapies in Cancer: Drug Nanocarriers, the Future of Chemotherapy. Eur. J. Pharm. Biopharm..

[62] Mignani S., Rodrigues J., Tomas T., Roy R., Shi X., Majoral J.-P. (2017). Bench-to-bedside translation of dendrimers: Reality or utopia? A concise analysis. Adv. Drug Deliv. Rev..

[63] Kesharwani P., Iyer A.K. (2015). Recent Advances in Dendrimer-Based Nanovectors for Tumor-Targeted Drug and Gene Delivery. Drug Discov. Today.

[64] Govender P., Therrien B., Smith G.S. (2012). Bio-Metallodendrimers—Emerging Strategies in Metal-Based Drug Design. Eur. J. Inorg. Chem..

[65] Jansen B.A.J., van der Zwan J., Reedĳk J., den Dulk H., Brouwer J. (1999). A Tetranuclear Platinum Compound Designed to Overcome Cisplatin Resistance. Eur. J. Inorg. Chem..

[66] Hurley A.L., Mohler D.L. (2000). Organometallic Photonucleases:  Synthesis and DNA-Cleavage Studies of Cyclopentadienyl Metal-Substituted Dendrimers Designed To Increase Double-Strand Scission. Org. Lett..

[67] Govender P., Antonels N.C., Mattsson J., Renfrew A.K., Dyson P.J., Moss J.R., Therrien B., Smith G.S. (2009). Anticancer Activity of Multinuclear Arene Ruthenium Complexes Coordinated to Dendritic Polypyridyl Scaffolds. J. Organomet. Chem..

[68] Zhao X., Loo S.C.J., Lee P.P.-F., Tan T.T.Y., Chu C.K. (2010). Synthesis and Cytotoxic Activities of chloropyridylimineplatinum(II) and chloropyridyliminecopper(II) Surface-Functionalized Poly(amidoamine) Dendrimers. J. Inorg. Biochem..

[69] Govender P., Renfrew A.K., Clavel C.M., Dyson P.J., Therrien B., Smith G.S. (2011). Antiproliferative Activity of Chelating *N*,*O*- and *N*,*N*-Ruthenium(II) Arene Functionalised Poly(propyleneimine) Dendrimer Scaffolds. Dalton Trans..

[70] Robilotto T.J., Alt D.S., von Recum H.A., Gray T.G. (2011). Cytotoxic Gold(I)-Bearing Dendrimers from Alkyne Precursors. Dalton Trans..

[71] Ahamad T., Mapolie S.F., Alshehri S. (2012). Synthesis and Characterization of Polyamide Metallodendrimers and Their Anti-Bacterial and Anti-Tumor Activities. Med. Chem. Res..

[72] Govender P., Sudding L.C., Clavel C.M., Dyson P.J., Therrien B., Smith G.S. (2013). The Influence of RAPTA Moieties on the Antiproliferative Activity of Peripheral-Functionalised Poly(salicylaldiminato) Metallodendrimers. Dalton Trans..

[73] Payne R., Govender P., Therrien B., Clavel C.M., Dyson P.J., Smith G.S. (2013). Neutral and Cationic Multinuclear Half-Sandwich Rhodium and Iridium Complexes Coordinated to Poly(propyleneimine) Dendritic Scaffolds: Synthesis and Cytotoxicity. J. Organomet. Chem..

[74] El Brahmi N., El Kazzouli S., Mignani S.M., Essassi E.M., Aubert G., Laurent R., Caminade A.-M., Bousmina M.M., Cresteil T., Majoral J.-P. (2013). Original Multivalent Copper(II)-Conjugated Phosphorus Dendrimers and Corresponding Mononuclear Copper(II) Complexes with Antitumoral Activities. Mol. Pharm..

[75] Sudding L.C., Payne R., Govender P., Edafe F., Clavel C.M., Dyson P.J., Therrien B., Smith G.S. (2014). Evaluation of the in Vitro Anticancer Activity of Cyclometalated Half-Sandwich Rhodium and Iridium Complexes Coordinated to Naphthaldimine-Based Poly(propyleneimine) Dendritic Scaffolds. J. Organomet. Chem..

[76] Govender P., Edafe F., Makhubela B.C.E., Dyson P.J., Therrien B., Smith G.S. (2014). Neutral and Cationic osmium(II)-Arene Metallodendrimers: Synthesis, Characterisation and Anticancer Activity. Inorg. Chim. Acta.

[77] Govender P., Riedel T., Dyson P.J., Smith G.S. (2015). Higher Generation Cationic *N*,*N*-ruthenium(II)-Ethylene-Glycol-Derived Metallodendrimers: Synthesis, Characterization and Cytotoxicity. J. Organomet. Chem..

[78] Maroto-Diaz M., Elie B.T., Gomez-Sal P., Perez-Serrano J., Gomez R., Contel M., Javier de la Mata F. (2016). Synthesis and Anticancer Activity of Carbosilane Metallodendrimers Based on Arene Ruthenium(II) Complexes. Dalton Trans..

[79] Michlewska S., Ionov M., Shcharbin D., Maroto-Díaz M., Gomez Ramirez R., Javier de la Mata F., Bryszewska M. (2017). Ruthenium Metallodendrimers with Anticancer Potential in an Acute Promyelocytic Leukemia Cell Line (HL60). Eur. Polym. J..

[80] Reagan M.R., Kaplan D.L. (2011). Concise Review: Mesenchymal Stem Cell Tumor-Homing: Detection Methods in Disease Model Systems. Stem Cells.

[81] Chang A.I., Schwertschkow A.H., Nolta J.A., Wu J. (2015). Involvement of Mesenchymal Stem Cells in Cancer Progression and Metastases. Curr. Cancer Drug Targets.

[82] Rhee K.-J., Lee I.J., Eom W.Y. (2015). Mesenchymal Stem Cell-Mediated Effects of Tumor Support or Suppression. Int. J. Mol. Sci..

[83] Jardim M.G., Rissanen K., Rodrigues J. (2010). Preparation and Characterization of Novel Poly(alkylidenamine) Nitrile Ruthenium Metallodendrimers. Eur. J. Inorg. Chem..

[84] Van Geelen C.M.M., de Vries E.G.E., Le T.K.P., van Weeghel R.P., de Jong S. (2003). Differential Modulation of the TRAIL Receptors and the CD95 Receptor in Colon Carcinoma Cell Lines. Br. J. Cancer.

[85] Tolan D., Gandin V., Morrison L., El-Nahas A., Marzano C., Montagner D., Erxleben A. (2016). Oxidative Stress Induced by Pt(IV) Pro-drugs Based on the Cisplatin Scaffold and Indole Carboxylic Acids in Axial Position. Sci. Rep..

[86] Klopp A.H., Gupta A., Spaeth E., Andreeff M., Marini F. (2011). Concise Review: Dissecting a Discrepancy in the Literature: Do Mesenchymal Stem Cells Support or Suppress Tumor Growth?. Stem Cells.

[87] Bartosh T.J., Ullah M., Zeitouni S., Beaver J., Prockop D.J. (2016). Cancer Cells Enter Dormancy after Cannibalizing Mesenchymal Stem/stromal Cells (MSCs). Proc. Natl. Acad. Sci. USA.

[88] Hong I.-S., Lee H.-Y., Kang K.-S. (2014). Mesenchymal Stem Cells and Cancer: Friends or Enemies?. Mutat. Res..

[89] Houthuijzen J.M., Daenen L.G.M., Roodhart J.M.L., Voest E.E. (2012). The Role of Mesenchymal Stem Cells in Anti-Cancer Drug Resistance and Tumour Progression. Br. J. Cancer.

[90] Rodrigues J., Jardim M.G., Figueira J., Gouveia M., Tomas H., Rissanen K. (2011). Poly(alkylidenamines) Dendrimers as Scaffolds for the Preparation of Low-Generation Ruthenium Based Metallodendrimers. New J. Chem..

[91] Bruce M.I., Windsor N.J. (1977). Cyclopentadienyl-Ruthenium and -Osmium Chemistry. IV. Convenient High-Yield Synthesis of Some Cyclopentadienyl Ruthenium or Osmium Tertiary Phosphine Halide Complexes. Aust. J. Chem..

[92] Blunden B.M., Stenzel M.H. (2015). Incorporating Ruthenium into Advanced Drug Delivery Carriers—An Innovative Generation of Chemotherapeutics. J. Chem. Technol. Biotechnol..

[93] Thangavel P., Viswanath B., Kim S. (2017). Recent developments in the nanostructured materials functionalized with ruthenium complexes for targeted drug delivery to tumors. Int. J. Nanomed..

