# Supplementary material for: Poly(alkylidenimine) Dendrimers Functionalized with the Organometallic Moiety [Ru(η5-C5H5)(PPh3)2]+ as Promising Drugs Against Cisplatin-Resistant Cancer Cells and Human Mesenchymal Stem Cells
Source: Molecules. 2018 Jun 17;23(6):1471. doi: 10.3390/molecules23061471 (PMC6100097; doi:10.3390/molecules23061471)
Supplement: Supplementary file 1 [file molecules-23-01471-s001.pdf]

molecules

Supplementary Material for

Poly(alkylidenimine) dendrimers functionalized with the organometallic moiety [Ru(η^5-C_5H_5)(PPh_3)$_2$]$^+$ as promising drugs against *cisplatin*-resistant cancer cells and human mesenchymal stem cells

Marisol Gouveia[1], João Figueira[2], Manuel G. Jardim[1], Rita Castro[1], Helena Tomás[1], Kari Rissanen[3] and João Rodrigues[1,4*]

[1] CQM - Centro de Química da Madeira, MMRG, Universidade da Madeira, Campus da Penteada, 9000-390 Funchal, Portugal; joaor@uma.pt; marisolgouveia5@gmail.com; mgjardim@uma.pt; ritacastro@uma.pt; lenat@uma.pt

[2] Department of Chemistry, ScilifeLab, Umeå University, KBC - building, Linnaeus väg 6, 90736 Umeå, Sweden; joao.figueira@umu.se

[3] University of Jyvaskyla, Department of Chemistry, P.O. Box. 35, FI-40014 Jyväskylä, Finland; kari.t.rissanen@jyu.fi

[4] School of Materials Science and Engineering/Center for Nano Energy Materials, Northwestern Polytechnical University, Xi'an, 710072, China;

* Correspondence: joaor@uma.pt or joaor@nwpu.edu.cn; Tel.: +351-291-705-100

Abstract: Here and for the first time, we show that the organometallic compound [Ru(η^5-C_5H_5)(PPh_3)$_2$Cl] (RuCp) has potential to be used as a metallodrug in anticancer therapy, and further present a new approach for the cellular delivery of the [Ru(η^5-C_5H_5)(PPh_3)$_2$]$^+$ fragment via coordination on the periphery of low-generation poly(alkylidenimine) dendrimers through nitrile terminal groups. Importantly, both the RuCp and the dendrimers functionalized with [Ru(η^5-C_5H_5)(PPh_3)$_2$]$^+$ fragments present remarkable toxicity towards a wide set of cancer cells (Caco-2, MCF-7, CAL-72, and A2780 cells), including cisplatin-resistant human ovarian carcinoma cell lines (A2780*cis*R cells). Also, RuCp and the prepared metallodendrimers are active against human mesenchymal stem cells (hMSCs), which are often found in the tumor microenvironment where they seem to play a role in tumor progression and drug resistance.

Keywords: dendrimers; nanocarriers; metallodrugs; ruthenium; platinum; *cisplatin*; cancer treatment; hMSCs; toxicity, nanomedicine.

1. Spectroscopic data (NMR and MS spectra)

1.1. Compound [{(η^5-C_5H_5)(PPh_3)$_2$Ru}$_4$(1)][CF_3SO_3]$_4$ **(3)**

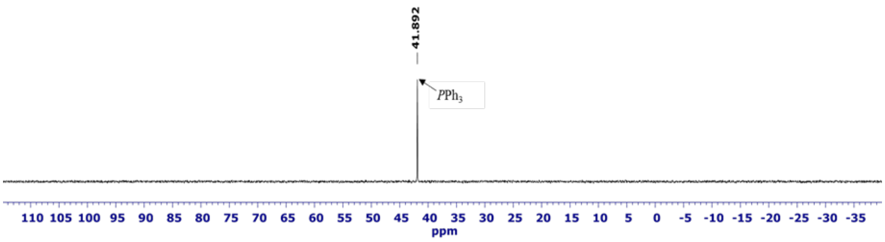

Figure S1. ^{31}P NMR spectrum of [{(η^5-C_5H_5)(PPh_3)$_2$Ru}$_4$(**1**)][CF_3SO_3]$_4$ (**3**), in $CDCl_3$.

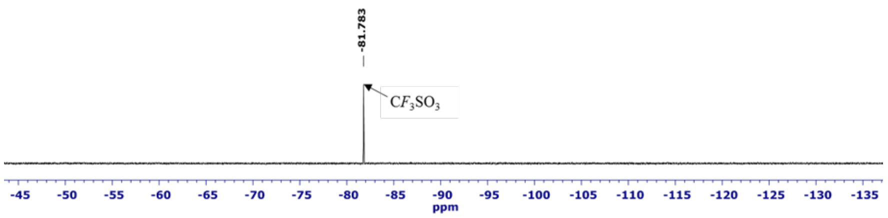

Figure S2. ^{19}F NMR spectrum of [{(η^5-C_5H_5)(PPh_3)$_2$Ru}$_4$(**1**)][CF_3SO_3]$_4$ (**3**), in $CDCl_3$.

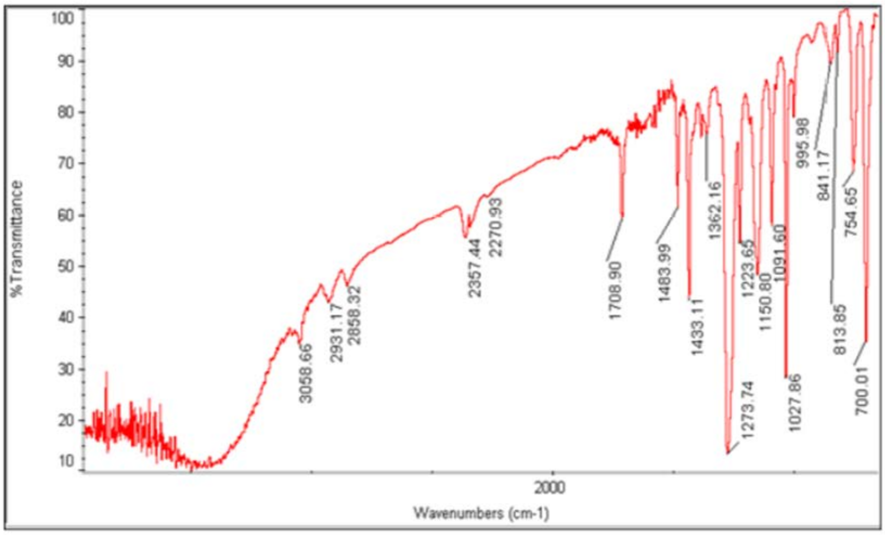

Figure S3. FTIR spectrum of [{(η^5-C_5H_5)(PPh_3)$_2$Ru}$_4$(**1**)][CF_3SO_3]4 (**3**), in KBr.

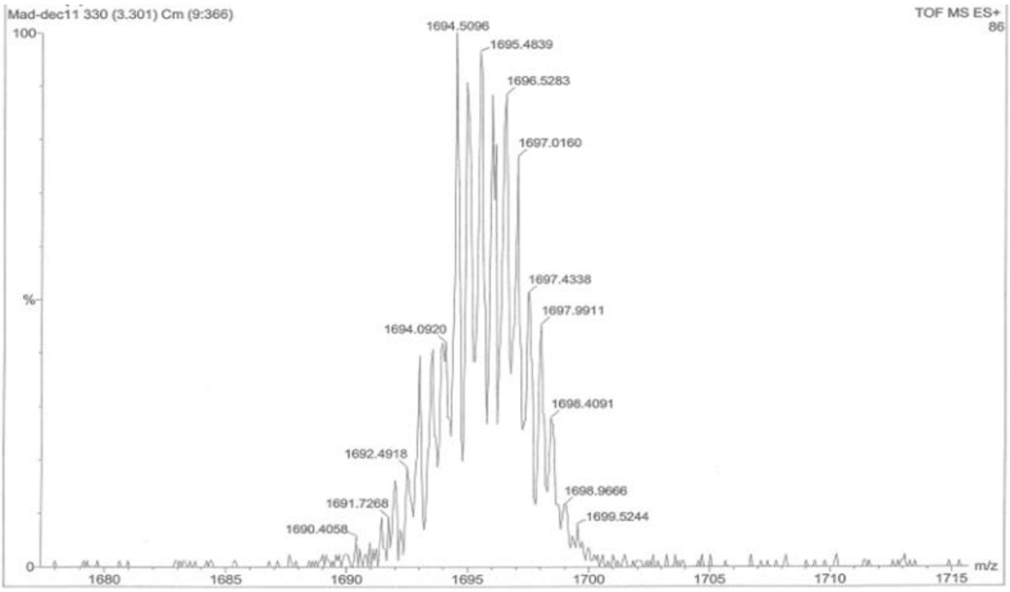

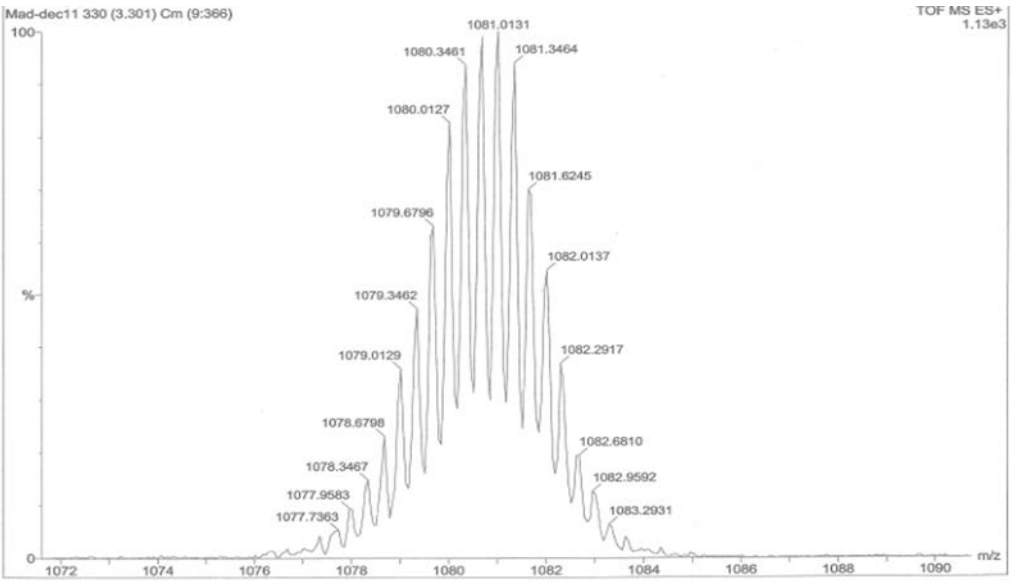

Figure S4. MS spectra of [{(η[5]-C5H5)(PPh3)2Ru}4(**1**)][CF3SO3]4 (**3**).

1.2. Compound [{(η[5]-C5H5)(PPh3)2Ru}4(2)][CF3SO3]4 **(4)**

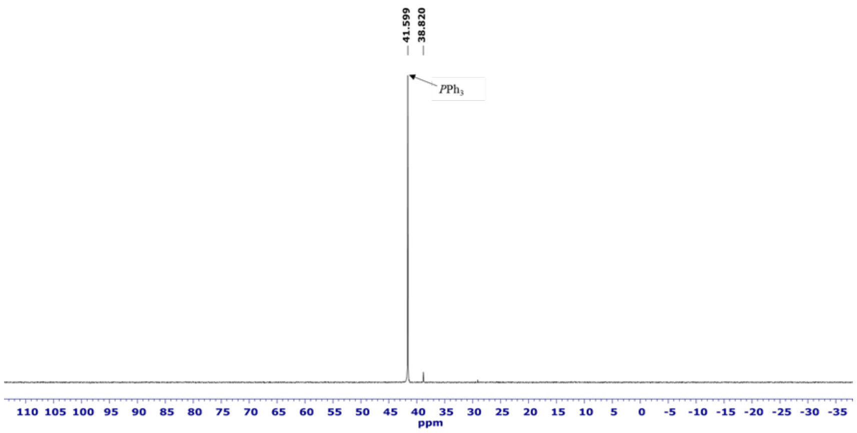

Figure S5. ^{31}P NMR spectrum of [{(η[5]-C5H5)(PPh3)2Ru}4(**2**)][CF3SO3]4 (**4**), in CDCl3.

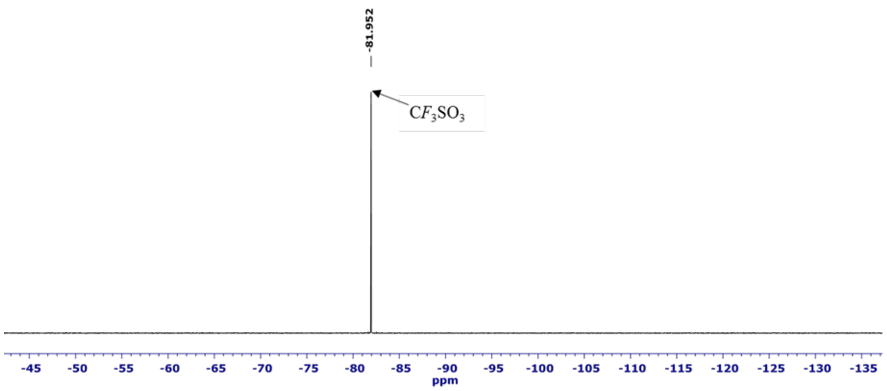

Figure S6. ^{19}F NMR spectrum of [{(η[5]-C5H5)(PPh3)2Ru}4(**2**)][CF3SO3]4 (**4**), in CDCl3.

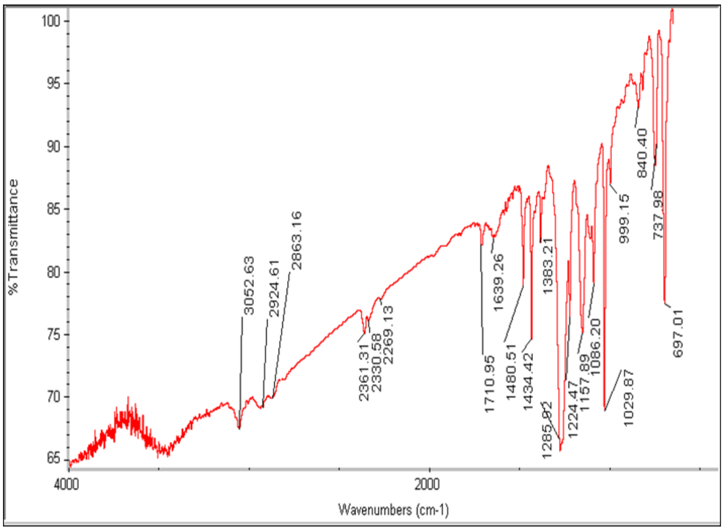

Figure S7. FTIR spectrum of [{(η^5-C_5H_5)(PPh_3)$_2$Ru}$_4$(**2**)][CF_3SO_3]$_4$ (**4**), in KBr.

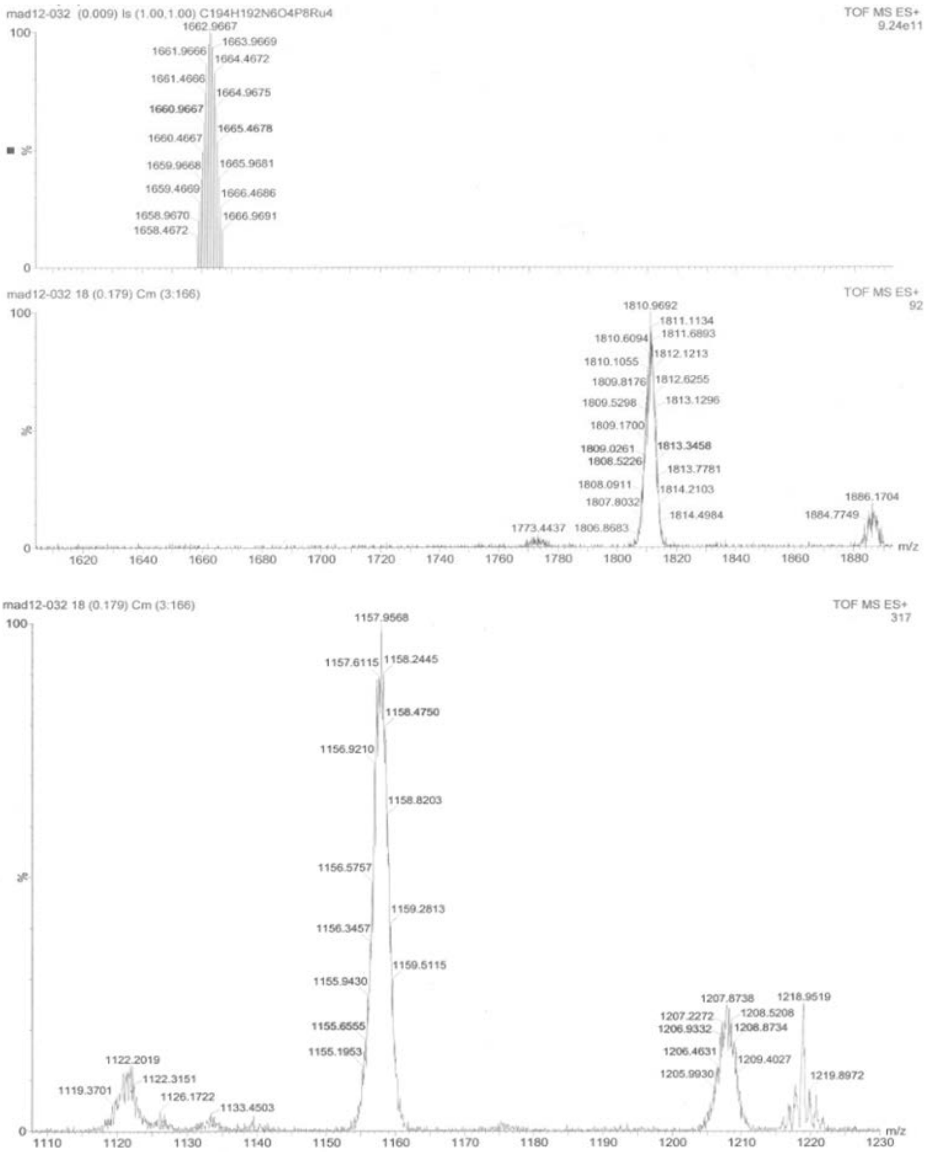

Figure S8. MS spectra of [{(η^5-C_5H_5)(PPh_3)$_2$Ru}$_4$(**2**)][CF_3SO_3]$_4$ (**4**).

1.3. PPh_3 compound

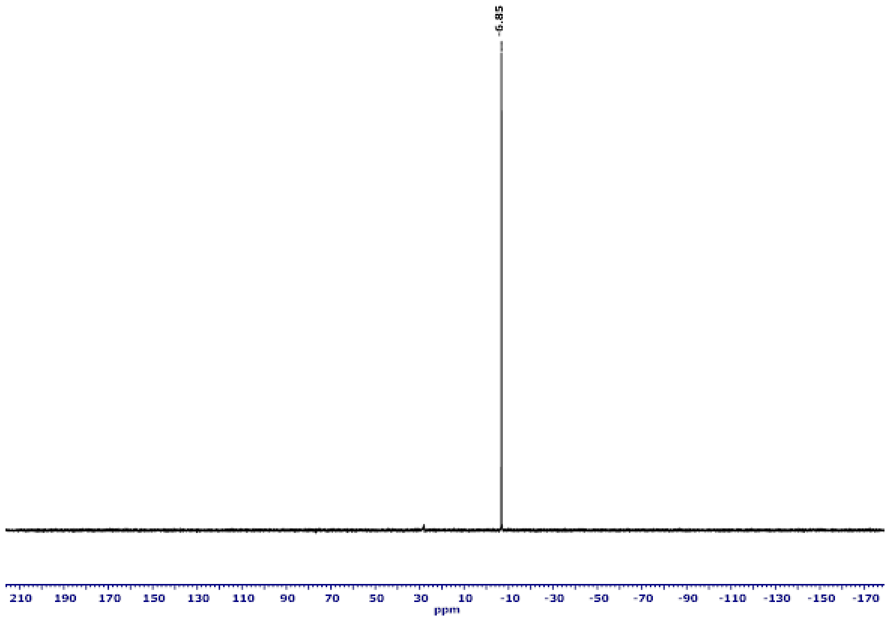

Figure S9. ^{31}P NMR spectrum of PPh_3 in a mixture of DMSO-d_6/D_2O.

2. Metabolic activity data

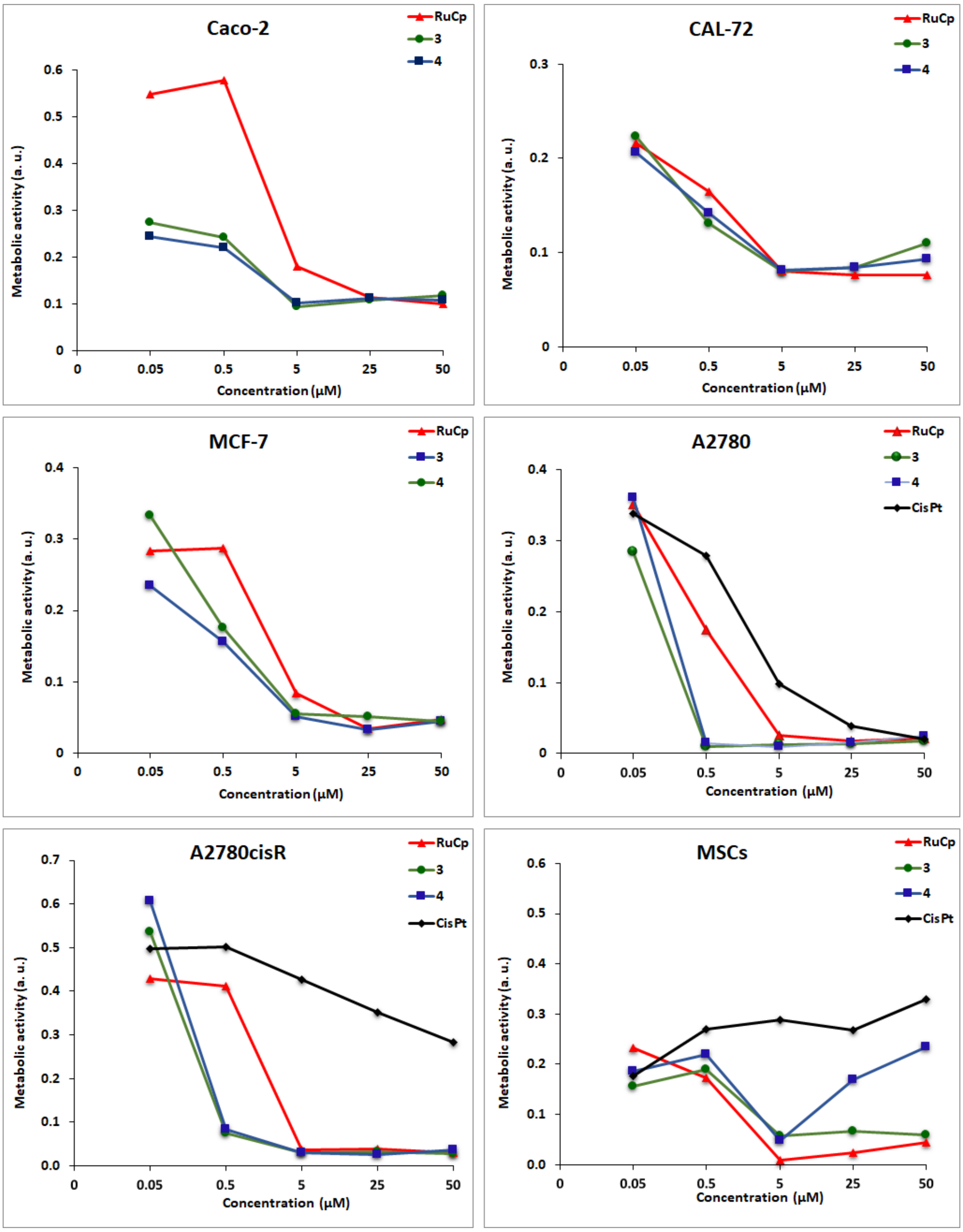

Figure S10. Effect of RuCp, metallodendrimers **3** and **4** and *cis*Pt on the metabolic activity (an indirect measure of cellular viability) of Caco-2, CAL-72, MCF-7, A2780, A2780*cis*R tumor cell lines, and on hMSCs.

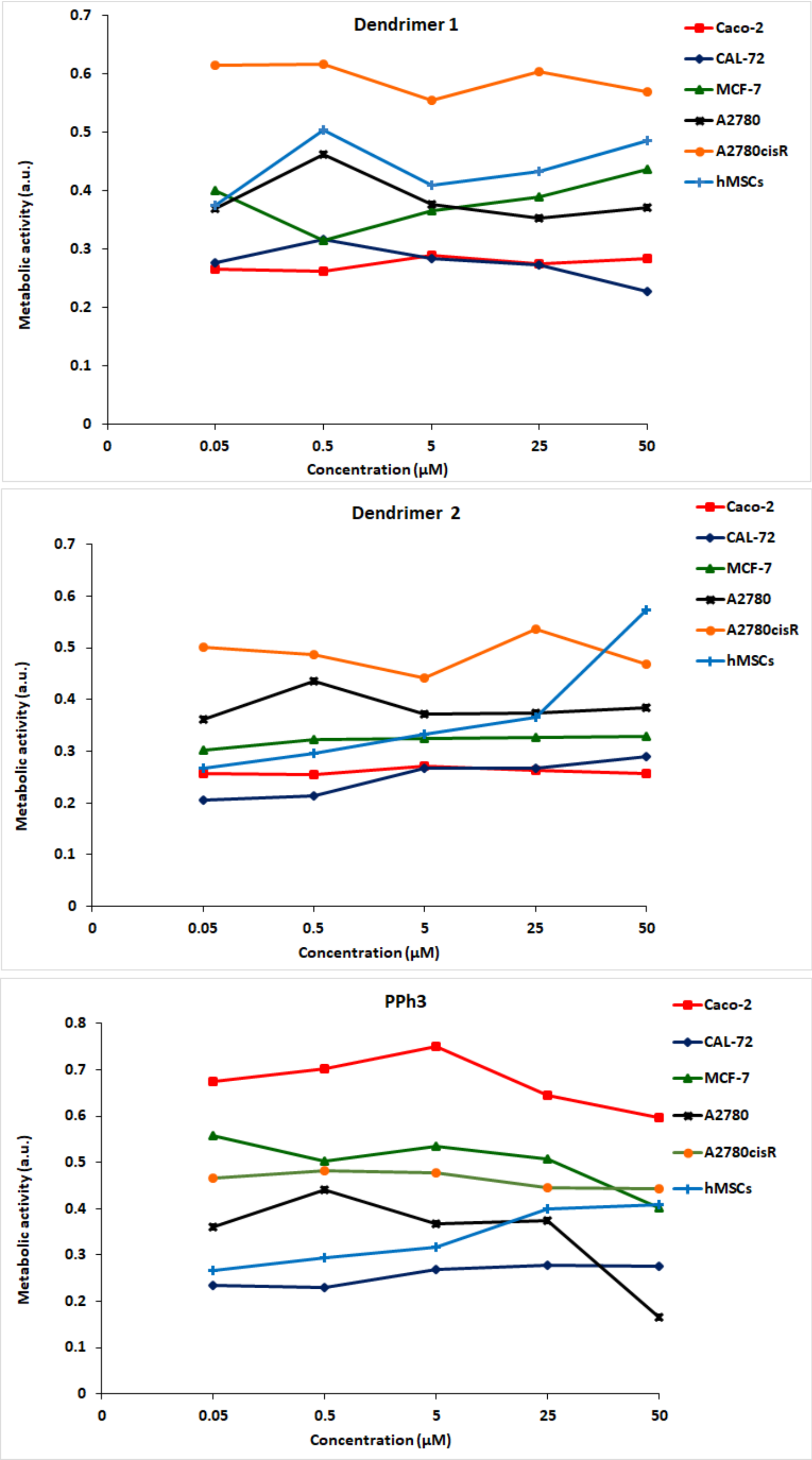

Figure S11. Effect of dendrimers **1** and **2** and PPh_3 on the metabolic activity (an indirect measure of cellular viability) of Caco-2, CAL-72, MCF-7, A2780, A2780*cis*R and hMSC cells.
